# Academic activism on behalf of children during the COVID-19 pandemic in Israel; beyond public health advocacy

**DOI:** 10.1186/s13584-021-00485-7

**Published:** 2021-08-18

**Authors:** Ora Paltiel, Hagit Hochner, David Chinitz, A. Mark Clarfield, Alex Gileles-Hillel, Amnon Lahad, Orly Manor, Ran Nir-Paz, Ari Paltiel, Chen Stein-Zamir, Ekaterina Yazhemsky, Ronit Calderon-Margalit

**Affiliations:** 1 Braun School of Public Health and Community Medicine, Hadassah Medical Organization, Faculty of Medicine, Hebrew University of Jerusalem, Jerusalem, Israel; 2 Faculty of Health Sciences, Ben-Gurion University of the Negev, Beer-sheva, Israel; 3 Faculty of Medicine, McGill University, Montreal, Canada; 4 Pediatric Pulmonology and Sleep Unit, Department of Pediatrics, Hadassah Medical Organization, Faculty of Medicine, Hebrew University of Jerusalem, Jerusalem, Israel; 5 Clalit Health Services, Jerusalem District, Israel; 6 Department of Clinical Microbiology and Infectious Diseases, Hadassah Medical Organization, Faculty of Medicine, Hebrew University of Jerusalem, Jerusalem, Israel; 7 School of Business Administration, Hebrew University of Jerusalem, Jerusalem, Israel

**Keywords:** COVID-19 in children, Child and adolescent health, School health, Public health policy, Public health advocacy, Academic activism

## Abstract

Among the challenges presented by the SARS-CoV2 pandemic are those related to balancing societal priorities with averting threats to population health. In this exceptional context a group of Israeli physicians and public health scholars (multidisciplinary academic group on children and coronavirus [MACC]) coalesced, examining the role of children in viral transmission and assessing the necessity and consequences of restricted in-class education. Combining critical appraisal and analytical skills with public health experience, MACC advocated for safe and monitored school re-opening, stressing the importance of education as a determinant of health, continuously weighing this stance against evolving COVID-19-risk data. MACC’s activities included offering research-based advice to government agencies including Ministries of Health, Finance, and Education. In a setting where government bodies were faced with providing practical solutions to both decreasing disease transmission and maintaining society’s vital activities, and various advisors presented decision-makers with disparate views, MACC contributed epidemiological, clinical and health policy expertise to the debate regarding school closure as a pandemic control measure, and adaptations required for safe re-opening. In this paper, we describe the evolution, activities, policy inputs and media profile of MACC, and discuss the role of academics in advocacy and activism in the midst of an unprecedented public health crisis. A general lesson learned is that academics, based on the rigor of their scientific work and their perceived objectivity, can and should be mobilized to pursue and promote policies based on shared societal values as well as empiric data, even when considerable uncertainty exists about the appropriate course of action. Mechanisms should be in place to open channels to multidisciplinary academic groups and bring their input to bear on decision-making.

## Background

Competing societal needs during the coronavirus pandemic have challenged policy-makers worldwide. Continuation or cessation of essential activities (eg. acute and preventive health services, cultural activities and commerce) were weighed against saving lives and mitigation measures to prevent healthcare system collapse. Around the world, decisions were made in a climate of uncertainty, governance was challenged, and public mistrust following frequent shifts in policy were observed in many countries. The pandemic prompted a deluge of research, but scientists also took on non-traditional roles in guiding policy.

School closures were among the commonest governmental responses to the pandemic, with Israel joining over 100 countries in instituting this measure. However, early on, uncertainty arose regarding the role of children in transmission of SARS-CoV2, the uniformity of disease susceptibility across age groups, alongside indications of severe social, health and economic consequences of school closures. Against this background, a multidisciplinary academic group (multidisciplinary academic group on children and coronavirus-MACC) crystallized in early April 2020 to inform policy and spur public debate on decisions affecting children in Israel.

The origins of the group were fortuitous. All had been present, as audience members or presenters, at a webinar on April 13, 2020, where early data from the pandemic were presented. The MACC group arose following an informal email exchange immediately after this webinar, questioning reports from China suggesting a limited role of children in SARS-CoV2 transmission [1], the publication of disease models assuming uniform transmission of the virus across age groups [2], and the appropriateness of influenza pandemic response and modelling for the current pandemic [3]. Through this correspondence all members of the group became involved in future activities. One member left after approximately 2 months, one stepped away temporarily, then rejoined, and one joined after three months.

The group aimed to review the available published and unpublished data regarding the role of children in the pandemic, analyze the risks and benefits of closures of educational facilities for children of all ages, advise policymakers and apprise the public about these issues, thus engaging in science-based public advocacy.

## Main text

### The Pandemic in Israel

Israel’s first case of COVID-19 was documented on February 21, 2020 and the first COVID-19-related death on March 20, 2020. The government took quick and drastic action to prevent transmission, morbidity and mortality, and avert a possible collapse of the healthcare system, including declaring a state of emergency, and rapid impositions of lockdowns, partial border and complete school closures. Each epidemic wave elicited various intensities of government response.

When detailed information about SARS-CoV2 transmission was still unavailable, many assumptions needed to be made. Models based on influenza and the SARS-CoV1 epidemic received early prominence in Israel and elsewhere [3]. Influenza, in contrast to COVID-19, is a disease in which the role of children and schools in transmission is important [4]. However, early school closure as an isolated epidemic control measure in the current SARS-CoV2 pandemic has not been tested rigorously for efficacy, and estimates of its potential impact show wide uncertainty, ranging from 20 to 40% mortality reductions [5], to provocative suggestions of mortality increases [6].

### Education, health, school closures and COVID-19

Education is not only a human right [7], but also a major determinant of health, associated with individual [8] and country-level higher life expectancy [9], higher childhood vaccination [10] and cancer survival rates [11]. In many countries, the education system contributes to reducing social inequalities as well as health disparities. Although the universal right to education is undisputed, the COVID-19 pandemic and its management have disproportionately disrupted children's lives and learning opportunities.

In contrast to the uncertain role of children in SARS-CoV2 transmission, the observed health effects of massive school closures globally have included increased levels of domestic violence [12], childhood obesity [13] as well as undernutrition [14], sleep disturbances [15], and psychological problems, mainly anxiety [16]. Moreover, school closures and the dependence on distance-learning increase educational and social gaps [17]. A modelling study in the US estimated that over 5 million years of life have been lost due to primary school closures during the pandemic [18].

### Israel: education system and school closures in response to COVID-19; timeline and response (Fig. 1)

**Fig. 1:**
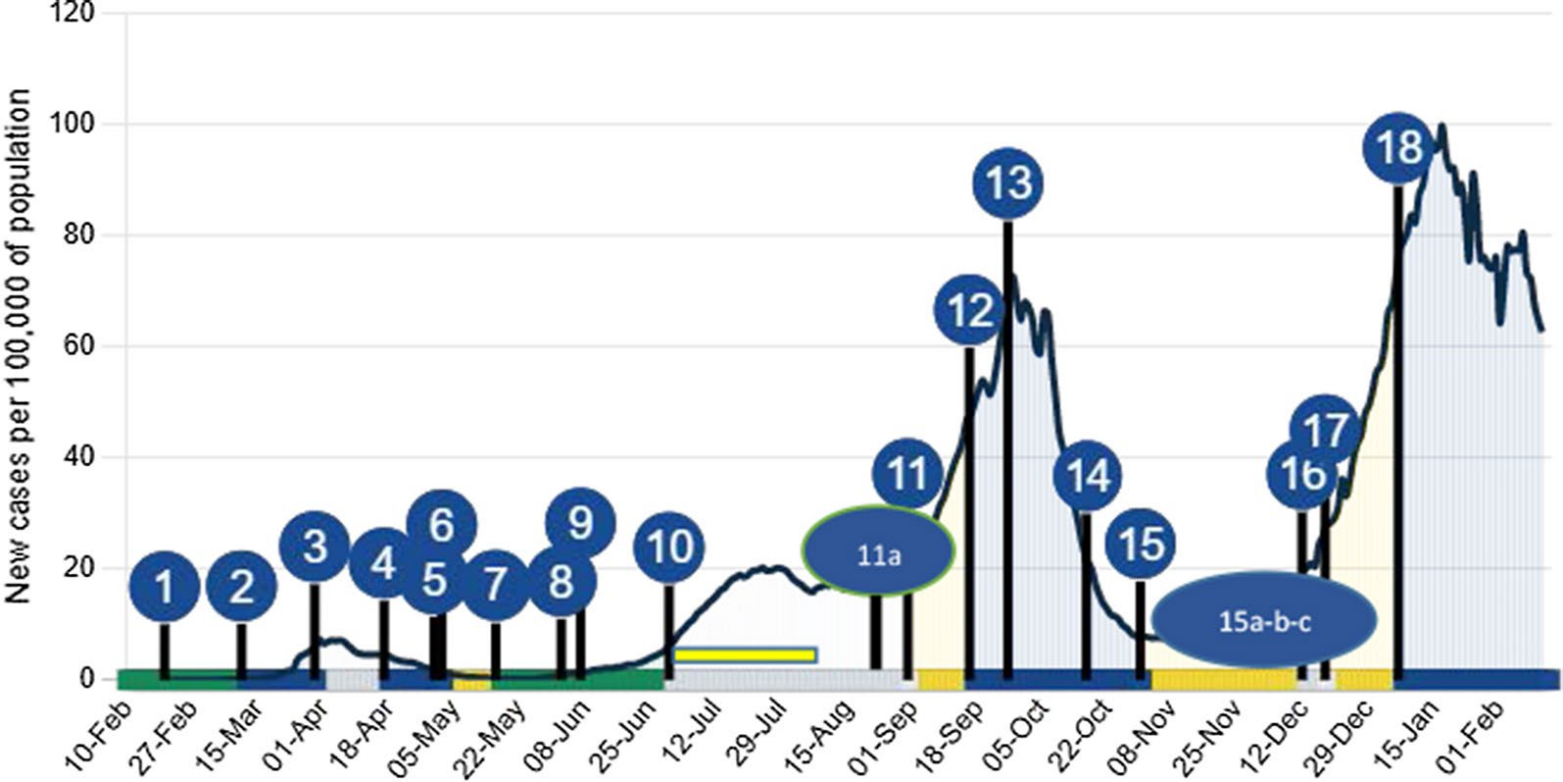
Timeline of school closure and reopening against the epidemic curve of SARS-CoV2 infection* in the Israeli population [52] (adapted and used with permission). (1) 21 Feb 2020 First COVID-19 cases reported. (2) 13 Mar Schools Closed (first national lockdown March 16). (3) 31 Mar Passover Vacation. (4) 18 Apr End of Passover vacation: School status changed to Closed. (5) 1 May Reopening announced. (6) 3 May Partially re-opening (grades 1–3 and 11–12 in-class teaching in “capsules”). Ultraorthodox schools (ages 13+) open due to pressure within and outside the government (7) 10 May Pre-schools reopened. On 17 May schools open in all sectors (without “capsules”, but with obligatory mask-wearing policy for age 7 and up.). (8) 3 Jun A new policy orders any school where a virus case emerges to close. (9) 8 Jun Closure of 100+ schools due to school outbreaks. (10) 21 Jun Middle and high schools closed. 1 Jul primary schools closed for summer vacation. Kindergarten to 3rd grade continued in optional summer school until August 8. (11a) 24 Aug Ultraorthodox schools (yeshivas) open. (11) 1 Sep Schools open except in high outbreak areas. (12) 17 Sep Nearly all schools close for 3-weeks due to rising infections. (2nd lockdown). (13) 27 Sep Peak of second wave. (14) 18 Oct Daycares, preschool and kindergarten for ages 0–6 reopen. (15) 1 Nov Schools partially open (grades 1–4 in "capsules"). (15a) 24 Nov Grades 5–6 return to school in “capsules” (cancelling capsules in grades 1–2). (15b) 29 Nov Grades 10–12 return to school. (15c) 6 Dec Grades 7–9 return to school. (16) 13 Dec Schools close Hannukah Vacation. (17) 19 Dec Schools partially open (kindergarten, Grades 1–4, 11–12). Other grades online exclusively. Third nationwide surge of infection and partial lockdown. 18) 8 Jan 2021 All schools closed, third general lockdown. *New daily cases per 100,000 of population, 7 day rolling average

Israel is a high-income country with the highest fertility rate per woman among OECD members [19]. Children aged ≤ 9 years comprise 19.7% of the population while youth aged 10–19 comprise a further 16.4% [20]. State-sponsored education is free from preschool at age three until completion of secondary education (12th grade). There are over 2.2 million children in pre-primary, primary and secondary schools, most receiving their education in the governmental system with some, mainly ultraorthodox, in the private sector [21]. There are two streams divided into four sectors, three Jewish (state, state-religious, and ultraorthodox Jewish) and one Arab (serving Muslim, Christian and Druze minorities), comprising 43%, 14%, 19% and 24% of children, respectively [20].

School-based proficiency exams reveal educational and infrastructure deficiencies in the Israeli school system. In the OECD Program for International Student Assessment (PISA) 2018, 15 year-old Israelis scored less than OECD average in reading literacy, mathematics and science, with considerable disparities among population groups [22]. Classroom crowding is also common; public secondary schools average 29 students per class (OECD average 23) [23] and many have 30 students or more [24], with physical classroom sizes smaller than recommended standards [24].

All educational facilities were closed on March 13, 2020, preceding a national lockdown. Subsequently, access to in-class education in Israel during the pandemic was among the lowest of OECD countries [25], especially for children in grades 5–10. Social distancing and mask-wearing were inconsistently enforced. Outbreaks of COVID-19 infection occurred sporadically in schools, including an occurrence at a Jerusalem middle and high school which began on May 26, following an extreme heatwave, during which the requirement for masks in classrooms was waived. The outbreak, which was quickly contained, involved 153 students with PCR-confirmed SARS-CoV2, with additional cases among teachers and family members [24].

**Fig. 2.**
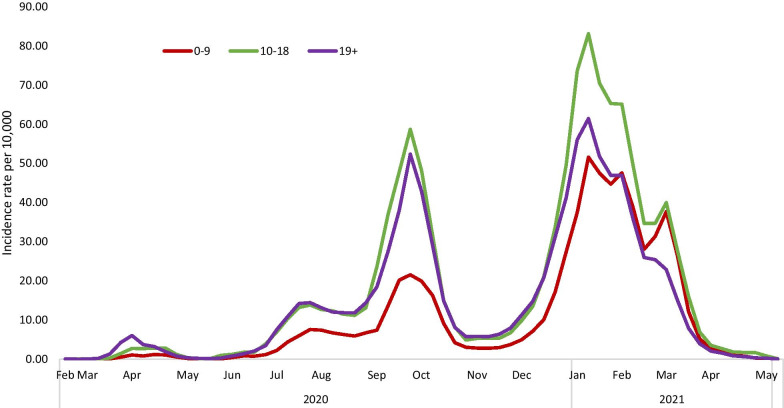
7-day Incidence rates of PCR confirmed SARS-CoV2 infection in Israel per 10,000, by age group 0–9, 10–18, 19+ years, by month, 2020–2021

Each epidemic wave brought increasing infections in children. The second wave was largely attributed to school reopening by the media and some Ministry of Health (MOH) officials. The third lockdown coincided with mass vaccination of Israelis aged 16+. The third wave, in which the B1.1.7 variant became predominant, was characterized by especially high rates of transmission to children, even during lockdown. SARS-CoV2 infections in children diminished dramatically following the mass vaccination of adults (Fig. 2). Vaccines were approved for youth aged 12–15 years on June 6 2021. This corresponded to the beginning of a fourth wave, comprising mainly the delta variant, and affecting young children disproportionately, due to the fact that they are not yet vaccinated. As of 30 July 2021, children aged 0–9 and youths aged 10–19 comprised 14.3% and 21.2% of the 869,063 confirmed coronavirus cases in Israel [26], respectively, while 11 deaths were reported among children aged ≤ 19, all with significant comorbidities.

### Public response to school closures and the origins of a public health response

Public protests in Israel to lockdown and COVID-19-control policies focused mainly on business closures, income loss, and rarely on schools. Exceptions were some secondary students, who staged sporadic low-key demonstrations demanding a return to in-class teaching. Gradually, parent organizations became involved in social media and media campaigns calling to resume in-class education.

Throughout the crisis, there was limited involvement by academic experts in the fields of education, child psychology and sociology in the public debate concerning school re-opening. However, high-profile government advisors frequently appeared in the media, referring to children as "the engine of the pandemic" and as "super-spreaders" [27], frequently noting that children with SARS-CoV-2 infection were mainly asymptomatic, unidentifiable and therefore posed a threat to the community.

The public debate in favor of school re-opening was spurred primarily by public health academics. As the pandemic progressed, the risks posed to children by a lack of structured learning and social interaction and the resultant threat of deepening social and health disparities, balanced against the generally mild effects of the disease itself, prompted MACC to take a stance advocating a cautious re-opening of schools for ages 1–10 while monitoring disease risks. This position contrasted with the government’s dominant focus, bolstered by risk-averse advisors, on the immediate health risks posed by SARS-CoV2, and the risks posed *by* children. As such, MACC’s strategy evolved from a neutral review of relevant scientific evidence and provision of information to policymakers and the public, to a more vigorously voiced ethical position regarding prioritizing education.

This report summarizes MACC’s activities, achievements in gaining access to decision-makers, igniting public debate regarding the role of children, and lessons learned during the COVID-19 crisis in Israel.

#### Group description

The MACC group included a diverse group of physicians with specializations in Public Health, Infectious Diseases, Pediatrics, Internal Medicine, Hematology, Family Medicine and Geriatrics; a biostatistician, epidemiologists, demographer, operations research analyst and health policy expert. All were teaching faculty of the Hebrew University, mainly in the Braun School of Public Health. MACC members, aged 40–70, held academic ranks ranging from instructor to full professor. The dozen members contributed strong quantitative skills in epidemiology and biostatistics, clinical experience, and a record of consultation and research in health policy.

Group members had taken on various professional roles during the pandemic, including community and hospital-based clinical care of COVID-19 patients, community epidemic control, COVID-related research published on a variety of pandemic-related topics [15, 24, 28–30], among others, mortality among Jewish populations worldwide, international comparison of mitigation measures in the first wave, crime statistics, public incentives and policing, and managing special funding for COVID-focused policy research. Group members also joined other national working groups producing and analyzing data.

#### Structure and communications process

There was no formal group leader; all decisions were made by consensus. Communication within MACC was via e-mail, telephone or a closed group messaging platform. Relevant literature, government reports and news items were shared often just minutes after events had occurred or publicly released by government ministries. Zoom meetings were held sporadically to develop strategy, especially preceding meetings with stakeholders and policymakers, and to assign tasks within the group. Requests for media appearances were fielded by seven of the twelve members. Meetings with government officials, all on virtual platforms, included 2–8 group members. Position papers and other written documents included input and were approved by all group members. Prior to formulating recommendations, the group held consultations with education experts in Israel.

#### Outputs

Table 1 outlines MACC’s advocacy activities and links to documents and activities.

**Table 1:** Advocacy activities on behalf of children and cautious school opening

Activity Year Month	Webinar/Conference	Position papers publicly released and letters to policy makers	Meetings with government officials
2020 April		*“Towards reopening kindergartens and elementary schools: A narrative review of COVID-19 infection, morbidity, and transmission in children”* https://corona-analysis.huji.ac.il/sites/default/files/coronaupdate/files/towards_reopening_schools_a_narrative_review_of_covid-19_infection_in_children_aged_1-10_years_10.6.20.pdf Communication of main findings from narrative review to the MOH*	MOF on return of children to schools
May	Webinar: *“Age in the age of corona”* https://huji.cloud.panopto.eu/Panopto/Pages/Viewer.aspx?id=41bbdaa8-b710-42b9-92ceabb300b13e8f&start=7.691007	Response to the Bnei Brak study to the Gertner Institute and MOH International comparison of schools return*	Head of Public Health Services, MOH
June		Recommendations for management of the educational system during the continuing COVID-19 pandemic—sent to MOH, MOE, MOF*	Director General of the MOH
July	Children and COVID, Nepal Pediatrician Association	Recommendations for safe return to school in the upcoming year – sent to the COVID project manager and MOE*	
August	Jewish Medical Society, UK Association of Americans and Canadians in Israel	Concerns on the plan to open the school year – addressed to the COVID project manager and the Head of the Knesset COVID Committee Recommendation not to reopen schools in areas of high community transmission addressed to COVID project manager, Heads of the Knesset COVID and Education Committees and MOE (recommendation accepted by government)	Director General of the MOH, and request for data regarding COVID-19 in children (granted) Education Minister on safe school opening and importance of maintaining “capsules” and small class sizes Chairwoman of Parliament’s COVID-19 Committee National Security Committee on reopening schools
September	Presentation to Physicians IMA International web-based conference: *“School opening in the corona era—where health, education, politics and religion meet"* https://huji.cloud.panopto.eu/Panopto/Pages/Viewer.aspx?id=30b0d377-0759-43d8-8168-ad6600684ab8		The National Center for Information and Knowledge on COVID-19
October	Presentation to physicians IMA Hospital Pediatric Rounds Jerusalem Conference, Israel Association of Family Physicians	Press release and detailed peer review of the MOH report on COVID-19 in children* Response to MOH serology survey* Position paper to return children up to age 10 to in-class schooling*	Presentation in the Parliament’s Education Committee and the COVID-19 committee Head of Public Health services, MOH Jerusalem Municipality COVID-19 education headquarters
November	Organization of Presidents of Teachers Colleges, Israel		Head of MOH Epidemiology Department Head of Maternal and Child health, MOH MOH COVID-19 project coordinator MOH and Minister of Science and Technology (thereafter two group members, OM and RCM, on advisory group to MOE) Jerusalem Municipality COVID-19 education headquarters The COVID-19 Minister Cabinet
December			Advisory Group to MOE
2021 January	Jerusalem Conference, Israel Association of Family Physicians	School reopening after the third wave*	MOH COVID-19 project coordinator
February		Open letter calling for sustainable program for in-class education addressed to Prime Minister, alternate Prime Minister and ministers of Health, Education and Finance*	
March	International web-based conference: *“SARS-CoV2, children and school closures, one year later—Estimating the risks and moving forward to sustainable solutions”* https://huji.cloud.panopto.eu/Panopto/Pages/Viewer.aspx?id=1dd92a77-deb0-42a8-9158-ad660067f845		

*MOH* Ministry of Health, *MOE* Ministry of Education, *MOF* Ministry of Finance, *IMA* Israeli Medical Association

^*^Documents are publicly available (in Hebrew) at: https://corona-analysis.huji.ac.il/updates-health

##### Narrative review

In April 2020, MACC conducted a narrative review of the current evidence of the role of children in the pandemic in four dimensions of COVID-19 in children: severity of disease, risk of infection, risk of transmission, and the effects of school closures, both on the pandemic as well as on children. At the time little was known about the subject. Initial clinical and epidemiological evidence came mostly from China, with some data on transmission to children from Iceland and Italy [31, 32], an early report on school-based transmission from Australia [33] and from modeling studies. We concluded, based on the available evidence, that children, especially those aged 1–10 years, were less likely to experience severe COVID-19 disease, appeared less likely to become infected than adults, were possibly less likely to transmit the disease, and that evidence suggesting that children were primary super-spreaders was lacking. Furthermore, the available literature did not provide proof that school closure was a highly effective intervention in curbing the epidemic, or that locations where primary schools had stayed open experienced major outbreaks among pupils or teachers.

##### Data analysis

The group analyzed Israeli MOH data regarding infection rates by age in children compared to adults, (see Fig. 2) and also compared rates by sex and population groups (not shown). Analysis revealed the relatively high rates of SARS-CoV2 infection in older teenagers, especially boys, and particularly those from the ultra-orthodox community.

##### Position papers, meetings, letters to policymakers

The findings of the narrative review, accompanied by specific recommendations, formulated after consultations with public health colleagues abroad (US, Canada Taiwan) were communicated to the MOH during the first wave. These included reopening preschool up to the 4rd grade in “capsules” (small pods/bubbles), while ensuring staggered entry and exit times and recesses, and enforcing increased hygiene measures as well as monitoring school outbreaks and infections in teachers and students. Subsequently, numerous interactions took place between MACC and policymakers, as well as with the media, and the group became publicly known as the “*Hebrew University experts on children and COVID*”. In meetings with policy makers, generally 2–8 members of the MACC group were present and meeting reports or minutes were sent to the entire group.

##### Responses to official reports

MACC was asked to provide methodologic input and pre-publication comments of reports prepared by official bodies. The first was of a government-commissioned study performed in the mainly ultra-Orthodox city of Bnei Brak on household transmission of SARS-CoV2 in children [34] which showed lower relative rates of secondary transmission to and from children, even in areas of high household density with large family sizes.

Secondly, MACC was invited to consult on a report on SARS-CoV2 transmission among children after school re-opening in September 2020, prepared by the National Center for Data and Knowledge for Combating Corona, an arm of Israeli Military Intelligence. MACC’s input included data interpretation and epidemiologic guidance.

In addition, the group independently appraised two important MOH reports. On 8/10/2020 a serologic study of Israelis undergoing laboratory testing in the health maintenance organizations was released [35, 36], reporting a 7% seroprevalence of IgG antibodies to SARS-CoV2 among children aged 7–19, compared to 3.8% overall. MACC’s critique noted that children had been under-sampled, along with critical population subgroups and geographic regions. In addition, children in the study were a biased toward a non-representative sub-group presenting to ambulatory clinics with a health complaint, whose parents agreed to have them undergo venipuncture. MACC concluded that the report could not be used for decision-making, since the true extent of past infection in Israeli children was impossible to discern from the data.

Perhaps the event which defined MACC’s activist role was its response to the Israel MOH report on SARS-CoV2 morbidity and transmission in children, released publicly on 18/10/2020 [37]. The report contained some previously unreported data, including detailed descriptions of COVID-19 symptoms experienced by children in various age groups, a description of super-spreader events in which children were involved, and a review of the epidemic curves by age and population group after school reopening in late August/early September. MACC’s response, while praising the wealth of detailed data, included the following critique:

i. The report focused on infection risks exclusively, disregarding the toll of school closures on children’s social and mental health and development, as well as their deleterious effects on the economy and in promoting inequalities.
ii. The report referred to “children” as one homogenous entity, while the data presented clearly demonstrated differential COVID-19 transmission patterns in young children aged 1–10 years, compared to teenagers, as well as by population sector.
iii. Most seriously, the report concluded that children could be super-spreaders, and as such school opening presented a danger to society as a whole, provoking headlines and public fear. In fact, the MOH’s own investigations revealed that children were infected *by* adults more often than infecting them, and actually constituted a small percentage (< 5%) of index-cases in super-spreader events.
iv. As noted, the serological data, based on a nonrepresentative sample could not be used to drive policy.

**Fig. 3:**
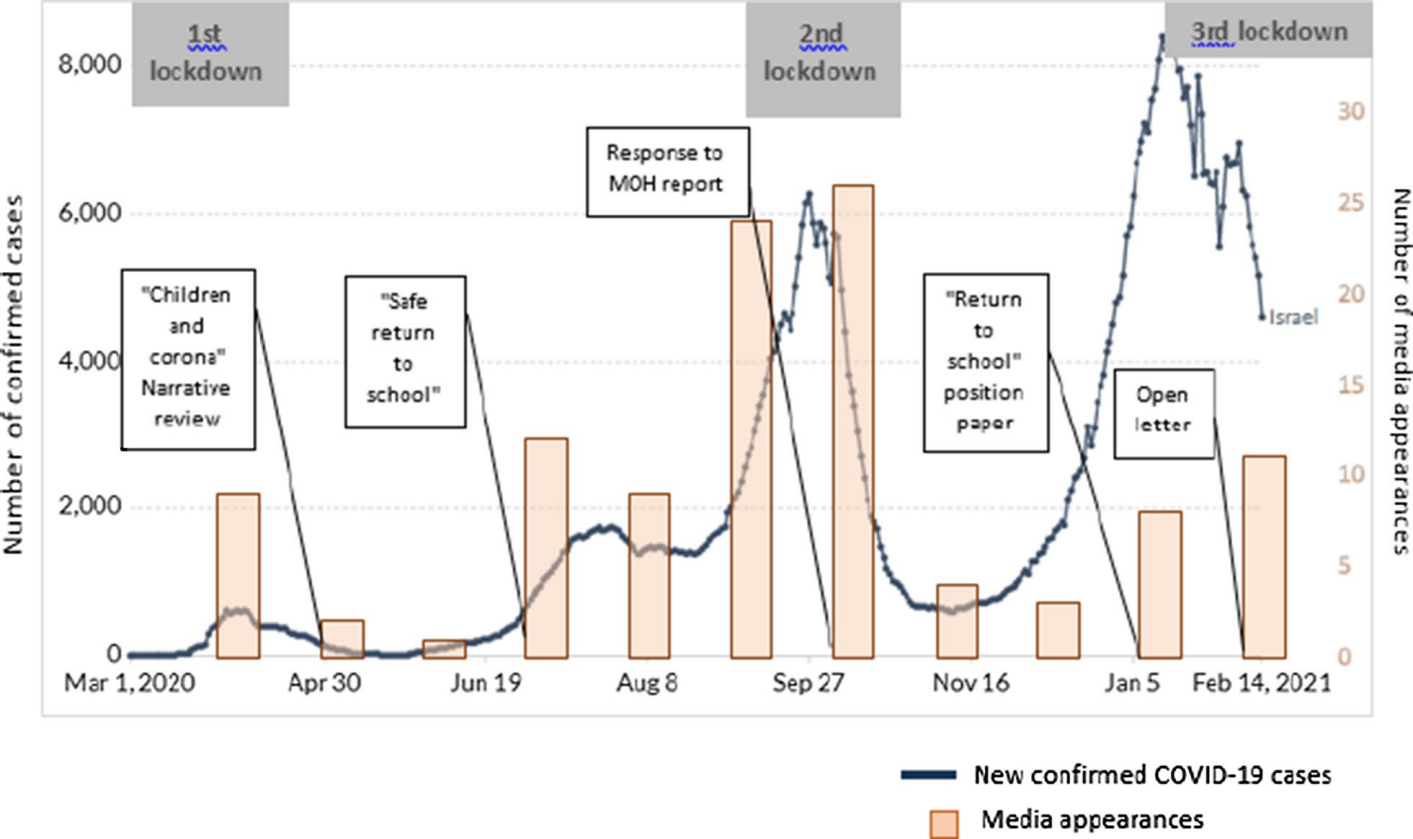
Media appearances of the MACC group related to COVID-19 and children. Columns represent media appearances in news channel sites, online press/newspapers, web news, broadcasts transcripts, over the period of April, 1, 2020 to February, 14, 2020 obtained via a comprehensive search in the Google search engine through explicit keyword searching in English and Hebrew ('children' + 'corona' + 'name of the researchers'). We discontinued the search process when at least 90% of the Google search page included irrelevant articles and we excluded duplicate articles. Grey boxes represent lockdown periods and white boxes represent position papers or press releases by MACC. Background solid line: confirmed COVID-19 cases from www.OurWorldInData.org licensed by Creative Commons, accessed 15/02/2021

While welcoming the wealth of information contained in the report, MACC described its tone and conclusions “biased and tendentious”, ascribing undue responsibility to children as propagators of the epidemic, rather than victims of its consequences. A press release on 21/10/2020 summarizing MACC's critique was widely reported and prompted many media appearances (Fig. 3). One week later, the group was invited to meet with the heads of the MOH Public Health Services, Epidemiology and Child Health branches to discuss the report in detail. This meeting, but especially the media attention, appeared to represent a turning point in MOH rhetoric regarding children. Rather than “super-spreaders” they became “contributors” to disease transmission. A change in language regarding education was also noted—the “value” of education was underscored, rather than merely emphasizing the dangers of in-class learning. This change in tone was also reflected in policy, as in the third wave school closures were the last policy measure enacted, after business closures and restrictions on public gatherings, rather than the first mitigation strategy, as had been the case in previous lockdowns.

Apart from direct interaction with national ministries, MACC also provided epidemiological consultation to the Jerusalem's municipality's headquarters for monitoring COVID-19 in schools, a local agency whose data management systems later served as a model of "best practice" for the rest of the country.

##### Public advocacy activities

i. Numerous webinars for local and, international audiences were held regarding the role of children in the COVID-19 epidemic. Presentations were also made to physicians’ organizations and professional societies.
ii. International publicly accessible web-based conferences. The first was held on September 23, 2020 (https://huji.cloud.panopto.eu/Panopto/Pages/Viewer.aspx?id=30b0d377-0759-43d8-8168-ad6600684ab8), attracting both the academic community and government officials. Besides MACC participants, invited speakers included a WHO representative, a prominent US school health consultant, the Israeli Health and Education ministries, head of the Israel parliamentary committee for education, a local mayor, head of the national parents’ committee, an academic researching the effect of school closure and online learning on children belonging to Israel’s Arab minority. The second was held on March 22, 2021 (https://huji.cloud.panopto.eu/Panopto/Pages/Viewer.aspx?id=1dd92a77-deb0-42a8-9158-ad660067f845), was co-sponsored by the MACC group and the Israel Pediatrics Society, Society for Pediatric Infectious Diseases and National Council for Community Health. Invited speakers included the head of the UK Royal College of Pediatricians, and the coordinator of school policy for Norway as well as Israeli pediatricians, psychiatrist, head of the Israel Council for the Child, and representatives of the Ministry of Health, including the director of its Public Health Services who served as discussant.
iii. Media appearances (Fig. 3).
  MACC members gave multiple interviews in the printed press, online publications, radio and television, podcasts, and briefed numerous journalists, both local and international, including the major Israeli networks. Some contributed essays to the printed press. These “appearances” were prompted either by Coronavirus-related events involving children, or followed publication and release of MACC reports and position papers via the University’s spokesperson. Figure 3 likely under-estimates the numbers of events, since group members were also asked about children during interviews on general COVID-19 issues, and in languages other than Hebrew and English.
iv. The MACC also consulted and collaborated with other formal and informal public bodies including the Israel Pediatric Society and parents’ organizations.

## Discussion and conclusions

The role of science and scientists in crafting policy is frequently debated. Tension exists between the preference for an arms-length relationship between the academy and government, where science proceeds in its search for "truth", unimpeded by the interests of politicians and partisan debate and the recognition that research priorities are value-laden, and cannot be dissociated from sectoral interests, including those of the scientists themselves. Politics can influence science and the research agenda [38]. Although science is expected to provide clear answers to guide public health policy, it almost always fails, because the consequences of recommended actions are uncertain, data are “messy”, spawning legitimate scientific disagreements in interpretation, and values are clearly implicated. An alternative, and probably more realistic expectation, is that scientists, especially public health scholars, contribute to policy according to an enlightenment model [39], recognizing the social context of the knowledge they present, as well as creating the “intellectual conditions for problem solving”.

In the particular context discussed herein, of children, school closures and COVID-19, the MACC group researched, uncovered and synthesized data, which emphasized not only case numbers, but also their meanings and implications for both health and the wider social context. This contrasted with messages conveyed by media and government officials that school closures were merely an economic impediment, without regard to their health consequences to children and families. MACC translated the imperfect knowledge regarding children, schools and COVID-19 into a clear but cautious recommendation to re-open schools gradually, along with appropriate monitoring and safety recommendations.

In accord with the enlightenment model, MACC was able to recommend various steps needed to re-open schools safely, but it could not offer a full model of how to budget for such efforts nor enforce them. Opening schools, like closing them, entails risks. The group made significant public contributions by presenting the available data regarding the relative benefit of reopening schools in areas of low transmission, pointing out the degree to which risks attributed to children were exaggerated, and suggesting that children were being demonized for causing viral transmission, whereas they were in essence victims of mitigation policies. All this was balanced by the recognition that COVID-19 indeed did pose *some* (albeit small) risk to children, and that infected, asymptomatic children posed some risk to vulnerable adults, including teachers. MACC consistently emphasized social distancing, hygiene and alleviation of school crowding as necessary public health measures, and once available, also encouraged vaccination of vulnerable populations and teachers. The group recognized the pressures experienced by overworked and harried government officials charged with ensuring the public’s health. These officials were influenced by risk-averse advisors, while simultaneously having to answer claims by other high-profile scientists and physicians, who played down the epidemic’s dangers on various popular media platforms. While sometimes opposing official government positions, MACC repeatedly offered and was called upon to assist officials with decision-making, and focused their information gathering and advocacy on a single issue (children and schools), while some other ad hoc groups tended to voice general opposition to imposed measures.

MACC's positions were largely in line with statements by professional pediatrics organizations (for example, in the UK [40], US [41] and Israel) relevant international bodies (WHO [42], Unicef [43]) and with other academics [44], with few exceptions [45]. The eventual acceptance by MOH officials that education is a “value”, and commitments to keep schools open despite rising SARS-CoV2 infections early in the third wave may or may not be causally attributed to MACC's activities. Nevertheless, the fact that government bodies continue to consult with MACC, demonstrates that the group is viewed as a credible and reputable source of information and counsel.

Public engagement is considered a core public health activity, and scientific activism has many precedents, including during the recent drinking water disaster in Flint, Michigan [46] as well as older examples, as remote as Virchow in the nineteenth century [47]. However, academic activism is also viewed with some suspicion. Thomas Wells, a British philosopher, argued that academic activism is “bad” for society, and an abuse of the trust that it holds in academics: “*In a civilized society, academic scientists are granted a special epistemic authority… This is because what they say about their subject of expertise is more likely to be true than what anyone else has to say about it. Unfortunately, some academics believe they have a right—or even a duty—to exploit this privileged status … for influencing society to do what they think best* [48]*.*”

Countering this position, Manchester University philosopher Anh Le wrote [49] “*In fact, many academics become activists because of what their research reveals. The nature of academic research is such that we are often in the position to come across many disturbing facts before the general public is aware of them. …When academics are in possession of these disturbing facts, it is their duty to promote these facts to the general public*"… and to "*fight injustice*".

While the academy focused on research and the influence of the COVID-19 pandemic on education (especially online learning), rare cries for academic engagement to influence policy were also heard. For instance, one university president advocated that [50]:

[academics should] engage more closely with policymakers and communicate our best insights to citizens and to the media in a clear and accessible manner in order to ensure that our research is informing democratic life and governance…

For the most part, members of the MACC group had not previously engaged in direct public health activism, other than in serving as members of government-appointed committees, or writing occasional op-eds or position papers. The COVID-19 crisis, described here, thus represented a paradigm change for MACC members: integrating their critical appraisal, data analysis and interpretation skills, and translating them into a clear ethical stance. This stance was informed by evidence, not merely on infection rates, but rather a holistic concept of health and a balanced view of risk.

There are a variety of lessons learned from the MACC experience. These include the recognition that training, experience and expertise arising from various disciplines, from community and hospital practice, from non-medical disciplines, and from age and gender diversity within the group, provide added value to decision-making in the face of uncertainty. The iterative and interactive process of rigorously examining the data available, both published and unpublished, by individuals from different professional backgrounds provided a fresh, broad perspective, perhaps less hampered by the “common wisdom” of each discipline, respectively. This multidisciplinary approach could serve as a model for inclusiveness and responsiveness of decision-makers, even between crises. Indeed, the group was generally well-received both by the media and by decision makers, even when in incomplete agreement with MACC’s recommendations.

The cautious approach characterizing MACC’s appearances in the media, with consistent messages based on examination of the evidence, could serve public health students in their training regarding advocacy. MACC’s activities were expedited by the relatively few ‘degrees of separation’ that typify Israeli society, enabling access to policymakers and the media. This lack of formality enhanced the group’s efforts and influence, and should facilitate future activism by academic groups, including public health professionals. However, barriers to this type of engagement may exist in countries with more structured and formal hierarchies. Nevertheless, a general lesson learned is that academics, based on the rigor of their scientific work and their perceived objectivity, can and should be mobilized to pursue and promote policies based on shared societal values as well as empiric data, even when considerable uncertainty exists about the appropriate course of action in a crisis.

For policy makers the lessons are even more important. Even during times of crisis there is opportunity to scan the policy landscape to seek and encourage groups in academia that may have the capacity for, and may have already begun to engage in multidisciplinary policy work relevant to decisions being made in real time. Mechanisms should be in place to open channels to such groups and bring their input to bear on decision-making.

In this type of public health crisis (which is not expected to be the last of its kind) authorities have a dual responsibility: both to provide guidance and mitigation measures, and to generate local data and information and integrate it into the global surveillance network. Faced with a novel disease, the benefits of sharing data as widely as possible are self-evident, but widening the circle for analysis and interpretation of these data are less accepted, challenging the standard mode of operation of government agencies. It can be expected that future post-COVID-19 public health crises will generate similar or greater levels of public-scientific engagement, and mechanisms should be developed to harness this rather than regard it as confrontational.

MACC’s activist approach is in keeping with the “all hands on deck” ethos of national, international and inter-sectoral collaboration to address COVID-19 and its consequences [51]. To paraphrase Hippocrates, “*Desperate times call for desperate measures*”. The COVID crisis generated an unusual multidimensional and multidisciplinary collaboration, thrusting a group of Israeli academics into an activist role, influencing policy while continuously monitoring the basis of their recommendations. As the pandemic unfolds, the role of children will eventually be clarified, but only by hindsight. COVID-19 presented a need for informed action before the dust settled.

Advocating for the educational rights of children in the health context is only a part of ensuring a secure future for Israel's post-COVID society. Future tasks include upgrading both the infrastructure and educational quality of the school system itself. The considerable challenges facing the system, even before the pandemic struck, belie Israel’s image as a “high tech” nation. Now more than ever, Israeli society must strive to make the necessary sustainable changes in order to promote the physical, mental and social welfare of its children.

## Data Availability

Not applicable.

## Abbreviation

MACC: Multidisciplinary academic group on children and coronavirus
